# Calcineurin/P-ERK/Egr-1 Pathway is Involved in Fear Memory Impairment after Isoflurane Exposure in Mice

**DOI:** 10.1038/s41598-017-13975-z

**Published:** 2017-10-24

**Authors:** Xiaoxuan Yang, Guohui Li, Qingsheng Xue, Yan Luo, Sensen Wang, Yimeng Xia, Lei Zhuang, Buwei Yu

**Affiliations:** Department of Anesthesiology, Ruijin Hospital, Shanghai Jiaotong University School of Medicine, Shanghai, 200025 China

## Abstract

Isoflurane exposure adversely influences subsequent fear memory formation in mice. Calcineurin (CaN), a phosphatase, prevents the establishment of emotional memory by dephosphorylating substrates and inhibiting the expression of learning and memory related genes. We investigated whether isoflurane impairment of fear memory formation was associated with altered CaN activity and downstream phosphorylated-extracellular signal-regulated kinases (p-ERK) and early growth response gene-1 (Egr-1) expression in hippocampus and amygdala. We also tested whether memory performance can be rescued by the CaN inhibitor FK506. Adult C57BL/6 mice were injected FK506 or vehicle after being exposed to 1.3% isoflurane or air for 1 h. After a 1 h- recovery, mice underwent classical fear conditioning (FC) training. Fear memory were tested 30 min, 48 h and 7 days after training. The activity of CaN, and expression of p-ERK and Egr-1 in hippocampus and amygdala were analyzed. Isoflurane exposure reduced mice freezing time in contextual and tone FC tests 30 min and 48 h after training. Hippocampus and amygdala from isoflurane-exposed mice had enhanced CaN activity, reduced p-ERK/ERK and Egr-1 expression. All these changes in isoflurane-exposed mice were attenuated by FK506 treatment. These results indicate calcineurin/p-ERK/Egr-1 Pathway is involved in fear memory impairment after isoflurane exposure in mice.

## Introduction

Both clinical and translational animal studies reveal that exposure to inhaled anesthetics can impair subsequent learning and memory. For example, after short surgical or diagnostic procedures with general anesthesia, up to 47% elderly patients demonstrated post-operative cognitive decline (POCD) at 24 h^1^. A pilot study by Zhang and colleagues^2^ suggested that isoflurane (Iso) might cause more cognitive dysfunction than desflurane. Adult rodents exposed to isoflurane display memory deficits that persist long after anesthetic elimination^3,4^. Identifying interventions to prevent POCD requires understanding of its mechanisms, which remain uncertain.

Calcineurin (CaN) also known as protein phosphatase 2B, is the only Ca^2+^- dependent Ser/Thr phosphatase in the brain^5^. CaN activity prevents fear memory formation in amygdala by dephosphorylating and inhibiting downstream kinases including ERK^6^. Egr-1 is a transcription factor that promotes expression of learning and memory related genes, positively modulating cognitive function^7^. Egr-1 is required for encoding new reference memory in hippocampus and new fear memory in the lateral amygdala^8,9^. Reul, *et al*. demonstrated that activation of ERK signaling pathways increased Egr-1 expression and facilitated memories of stressful events^10^. Furthermore, CaN inhibition in the hippocampus or amygdala is associated with increased expression of Egr-1 and memory enhancement^11,12^. This evidence strongly suggests that the CaN/p-ERK/Egr-1 signaling pathway is involved in the formation of emotional memory.

We hypothesized that isoflurane produces sustained impairment of emotional memory formation by enhancing CaN activity, subsequently decreasing ERK phosphorylation and Egr-1 expression. To initially test this hypothesis, we used a classical fear conditioning (FC) paradigm, including a hippocampus-dependent context fear memory test and an amygdala-dependent tone memory test^13^. We investigated the influence of isoflurane exposure on subsequent short-term and long-term fear memory formation and the CaN/p-ERK/Egr-1 signaling pathway in both hippocampus and amygdala. We also tested whether the CaN inhibitor FK506 could reverse isoflurane-induced fear memory impairment.

## Results

### Isoflurane blocked short-term and long-term fear memory

**Figure 1:**
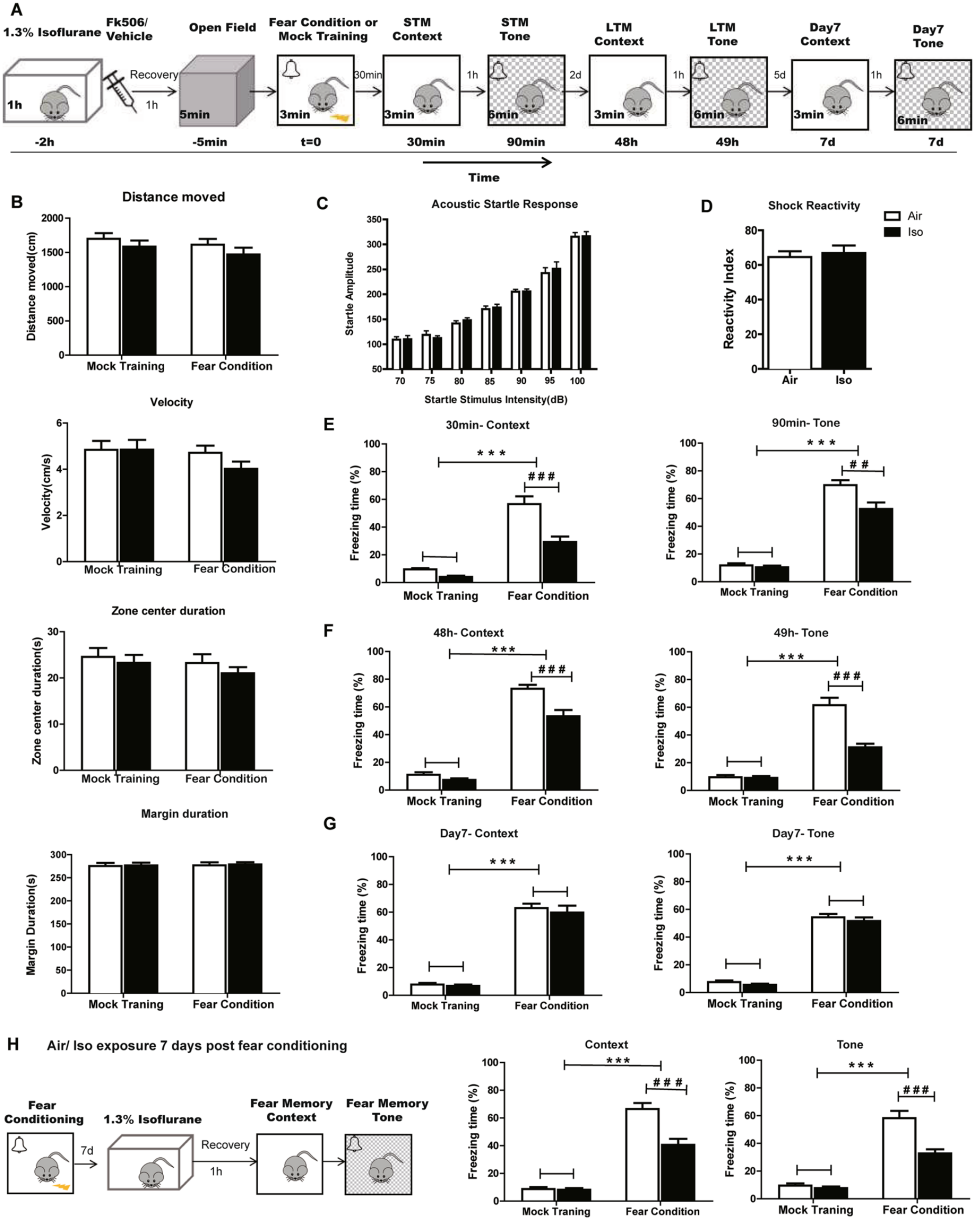
Experimental paradigm timeline and the influence of isoflurane exposure on mice fear memory. (**A**) Experimental paradigm timeline. The duration of each stage is inset at the bottom left. Interval time is indicated between the boxes. Relative time is indicated below the boxes (t = 0, start of fear conditioning training). Before fear conditioning, mice are exposed to 1.0 MAC isoflurane or 30% oxygen for 1 h followed by injection with FK506 or vehicle. Five min before fear conditioning, mice are examined in the open field. Then short-term memory (STM) and long-term memory(LTM) of the context and tone are tested 30 min and 48 h after conditioning, respectively. Seven days post conditioning, the same memory tests are used to observe how long the memory blockade effect of isoflureane can last. (**B**) Mice from all groups showed similar distance, move velocity, center and margin duration time in a 5 min-open field test before fear condition training. (**C**) Acoustic startle response tested after 1 h recovery from air/Iso exposure: mice from Air group and Iso group had similar startle response to varying acoustic stimuli. (**D**) The reactivity to electric foot shock did not differ among Air and Iso group. (**E~G**) show the freezing time (%) of each group during context and tone fear memory test. (**H**) Experimental paradigm timeline and freezing time (%) of mice which were exposed to Air/Iso 7 days post fear conditioning. n = 14 for each group. ***P < 0.001 compared with mock training group; ^###^ *P* < 0.001 compared with Iso group.

Figure 1A summarized the time course of the experimental procedures. First of all, we investigated the stability of our fear conditioning model. To eliminate the possibility that isoflurane modifies the behavior of mice, including motor ability and anxiety which could confound studies of fear memory, an additional 5 min-open field test was performed before fear condition training. During the induction of isoflurane anesthesia, the latency of LoRR was measured and compared between mock training (MT) and FC group. There was no significant difference of latency between MT and CFC group (150.2 ± 14.2s V.s 144.1 ± 14.6 s, n = 12 per group). As shown in Fig. 1B, distance and move velocity were not influenced by 1.3% isoflurane exposure in both mock training groups and FC groups. The center and margin duration, which usually reflect the anxiety level of animals, also did not differ among all the groups.

In addition, to ensure that isoflurane is not altering perception of auditory CS and shock US, acoustic startle response was tested at the end of 1 h recovery from air/Iso exposure using a separate group of mice. Shock reactivity was also compared between Air group and Iso group. As shown in Fig. 1C, after 1 h recovery from isoflurane exposure, mice from Air group and Iso group had similar startle response to varying acoustic stimuli. The reactivity to electric foot shock also did not differ among Air and Iso group (Fig. 1D).

As shown in Fig. 1E, the freezing time (%) of FC groups, both Air and Iso, were obviously higher compared with mock training groups (Two-way ANOVA, Context: F = 107.5, *P* < 0.001; Tone: F = 258.1, *P* < 0.001), indicating the success of establishing a fear conditioning model. Then we evaluated the overall effects of isoflurane on short-term memory (STM), which was detected 30 min after FC training. Isoflurane exposure had no significant effect on short-term fear memory of mock training group mice as compared to air treatment (*P* > 0.05). However, mice subjected to FC after a 1 h isoflurane exposure exhibited context and tone memory deficits evidenced by lower freezing time (%) compared with air-treated FC controls (Two-way ANOVA, Context: F = 22.09, *P* < 0.001; Tone: F = 8.862, *P* < 0.001). For long-term memory (LTM), which was measured 48 h after conditioning, the Iso group of mice with FC training showed significantly decreased percent of freezing comparing with the air group of FC mice (Two-way ANOVA, Context: F = 18.83, *P* < 0.001; Tone: F = 24.39, *P* < 0.001, Fig. 1F; n = 14 per group), suggesting a blockade of long term context and tone fear memory. However, isoflurane had no significant effect on long-term fear memory of mock training group mice(*P* > 0.05). To observe the duration of isoflurane induced fear memory deficit, we tested a separate group of mice at day 7 to avoid the influence of multiple tests on fear memory, in which the freezing time (%) of FC groups still higher than mock training groups (Two-way ANOVA, Context: F = 288.34, *P* < 0.001; Tone: F = 595.06, *P* < 0.001, Fig. 1G), indicating FC trained mice still maintain the fear memory, while the memory blockade effect of isoflurane had disappeared.

The fact that the Iso and Air groups showed equivalent levels of freezing after 7 days, but not after 48/49 h, suggested that isoflurane affected memory expression. In order to specify which stage of fear memory was impaired by Iso exposure, we conducted the same behavior experiment but shifted Air/Iso exposure time point to 7 days post fear conditioning. Fear memory tests were performed after 1 h recovery from Air/Iso exposure. As shown in Fig. 1H, the Iso group of mice with FC training showed significantly decreased percent of freezing comparing with the air group of FC mice (Two-way ANOVA, Context: F = 16.88, *P* = 0.0002; Tone: F = 18.72, *P* < 0.001, Fig. 1H; n = 14 per group), suggesting fear memory expression was blockade by isoflurane exposure.

### Calcineurin activities increased in both hippocampus and amygdala after isoflurane exposure

**Figure 2:**
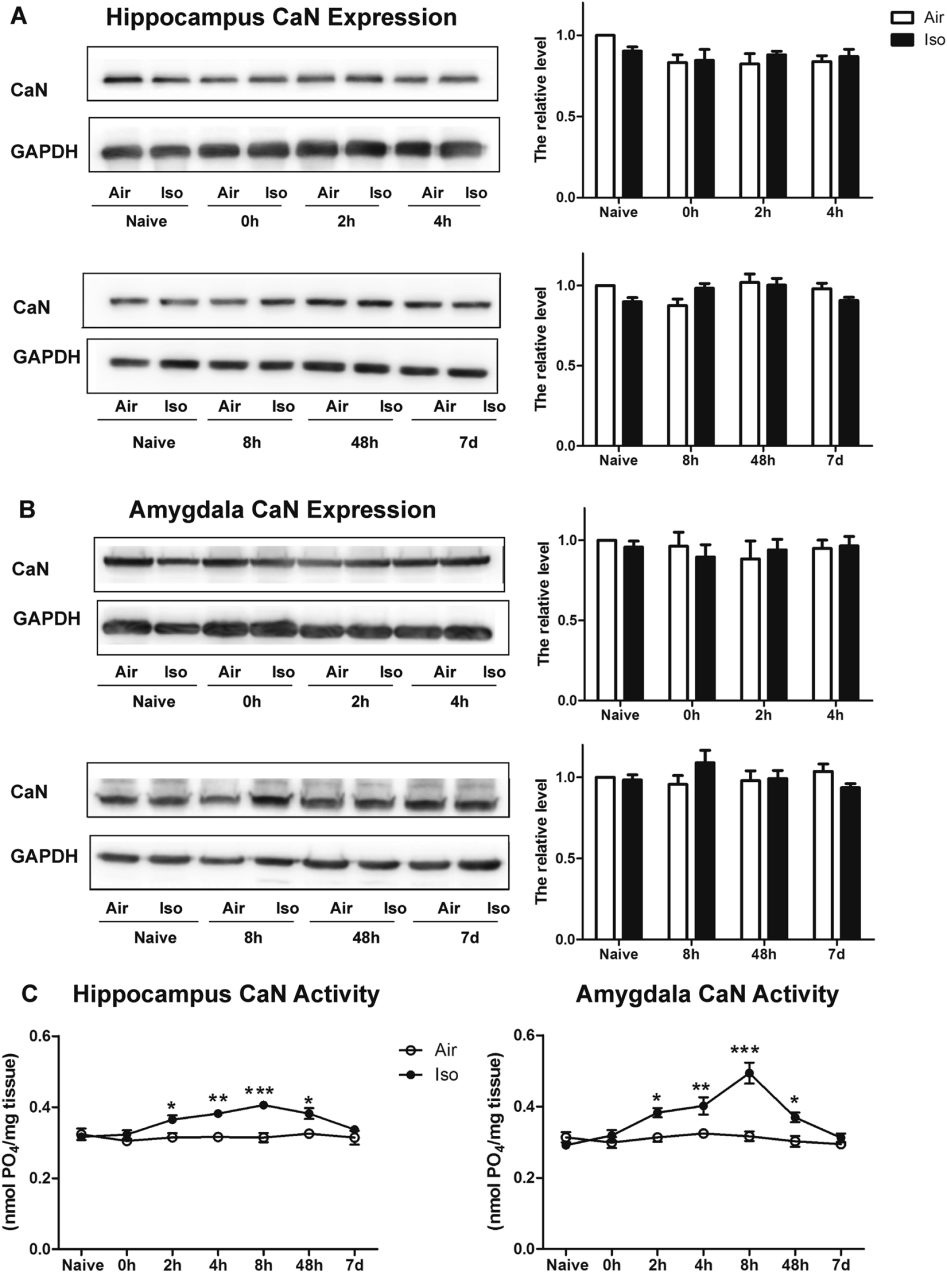
The influence of isoflurane on CaN in hippocampus and amygdala. No change of CaN protein expression is detected at different time points post isoflurane exposure in hippocampus (**A**: *P* > 0.05) and Amygdala (**B**: *P* > 0.05). Results were normalized against the mock training + Air group. The dephosphorylation efficacy of CaN, is markedly increased by isoflurane, it increased at 2 h after exposure, peaked at 8 h, persisted in a high level at 48 h, and went down to air group level 7 days after isoflurane exposure in both hippocampus and amygdala (**C**). *P < 0.05, ***P < 0.01, ***P < 0.001 compared with Iso group. n = 5~7 for each group.

In order to investigate whether CaN participated in the isoflurane-induced fear memory deficits, we detected its protein expression levels in both hippocampus and amygdala at 0 h, 2 h, 4 h, 8 h, 48 h and 7d post isoflurane exposure. We found that the expression levels of CaN in the Iso group were comparable to that in the Air group in both hippocampus and amygdala at any time point (Two-way ANOVA, Fig. 2A,B). However, the dephosphorylation activity of CaN significantly increased in both hippocampus and amygdala after isoflurane exposure, while those of the respective air groups didn’t change at any examined time point. Specifically, it increased at 2 h after exposure, peaked at 8 h, persisted in a high level at 48 h, and went down to air group level at 7d after exposure (Two-way ANOVA Bonferroni post-tests, Hippocampus: 2 h t = 2.858, P < 0.05, 4 h t = 3.765, *P* < 0.01, 8 h t = 5.227, *P* < 0.001, 48 h t = 3.239, *P* < 0.05; Amygdala: 2 h t = 3.133, *P* < 0.05, 4 h t = 3.471, *P* < 0.01, 8 h t = 7.949, *P* < 0.001, 48 h t = 3.013, *P* < 0.05, Fig. 2C), indicating an enhancement of its negative-regulation effects.

### Isoflurane exposure suppressed Egr-1 and P-ERK activity in both hippocampus and amygdala

**Figure 3:**
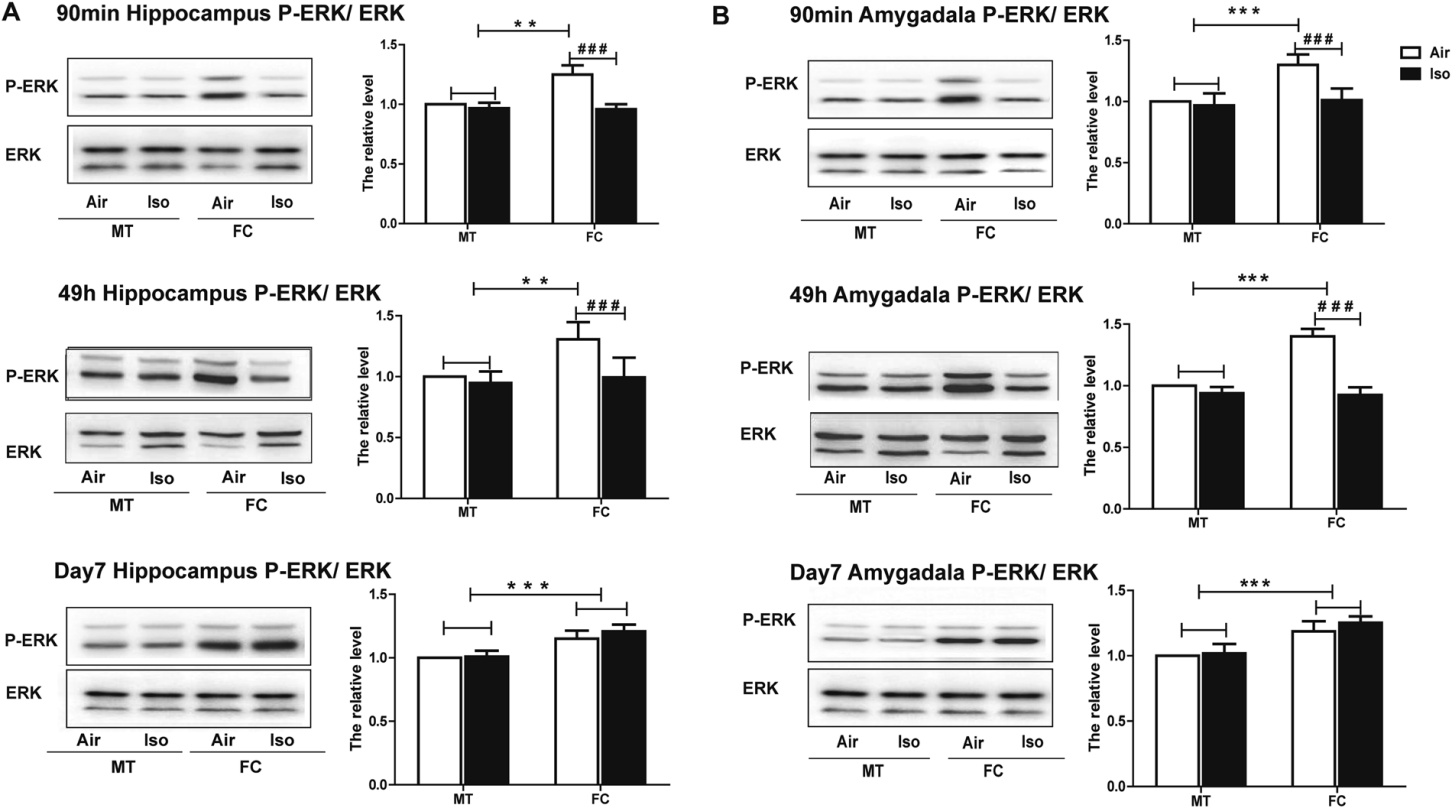
1 h isoflurane exposure decreased the phosphorylation of ERK stimulated by fear condition. (**A**) In hippocampus, compared to MT groups, FC groups showed significantly higher level of P-ERK 90 min, 49 h, 7 days post conditioning tone fear memory tests, which were impaired by isoflurane. 7 days after FC training, the inhibited ERK phosphorylation in FC group recovered to the similar level as Air group. (**B**) In amygdala, similar adverse effect of isoflurane on P-ERK can be seen. All data are presented as mean ± SEM, n = 6 for each group. **P < 0.05 compared with MT group; ***P < 0.001 compared with MT group; ^###^ *P* < 0.001 compared with Iso group.

After each 90 min, 49 h and 7 days post conditioning tone fear memory test, mice were decapitated and the hippocampus and amygdala were extracted to detect ERK phosphorylation and Egr-1 expression levels. As shown in Fig. 3, FC training increased ERK phosphorylation level in both hippocampus (Two-way ANOVA, 90 min post condition: F = 36.10, *P* < 0.0001; 49 h post condition: F = 13.48, *P* = 0.0016; 7 days post condition: F = 81.95, *P* < 0.0001 Fig. 3A) and amygdala (Two-way ANOVA, 90 min post condition: F = 26.66, *P* < 0.0001; 49 h post condition: F = 89.23, *P* < 0.0001; 7 days post condition: F = 77.63, *P* < 0.0001 Fig. 3B) compared with MT mice. However, this ERK phosphorylation significantly decreased 90 min and 49 h after FC training in both hippocampus (Two-way ANOVA, 90 min post condition: F = 63.98, *P* < 0.0001; 49 h post condition: F = 14.35, *P* = 0.0012, Fig. 3A) and amygdala (Two-way ANOVA, 90 min post condition: F = 23.63, *P* < 0.0001; 49 h post condition: F = 170.3, *P* < 0.0001, Fig. 3B). 7 days after FC condition, the inhibited ERK phosphorylation in FC group recovered to the similar level as Air group (Fig. 3A,B).

**Figure 4:**
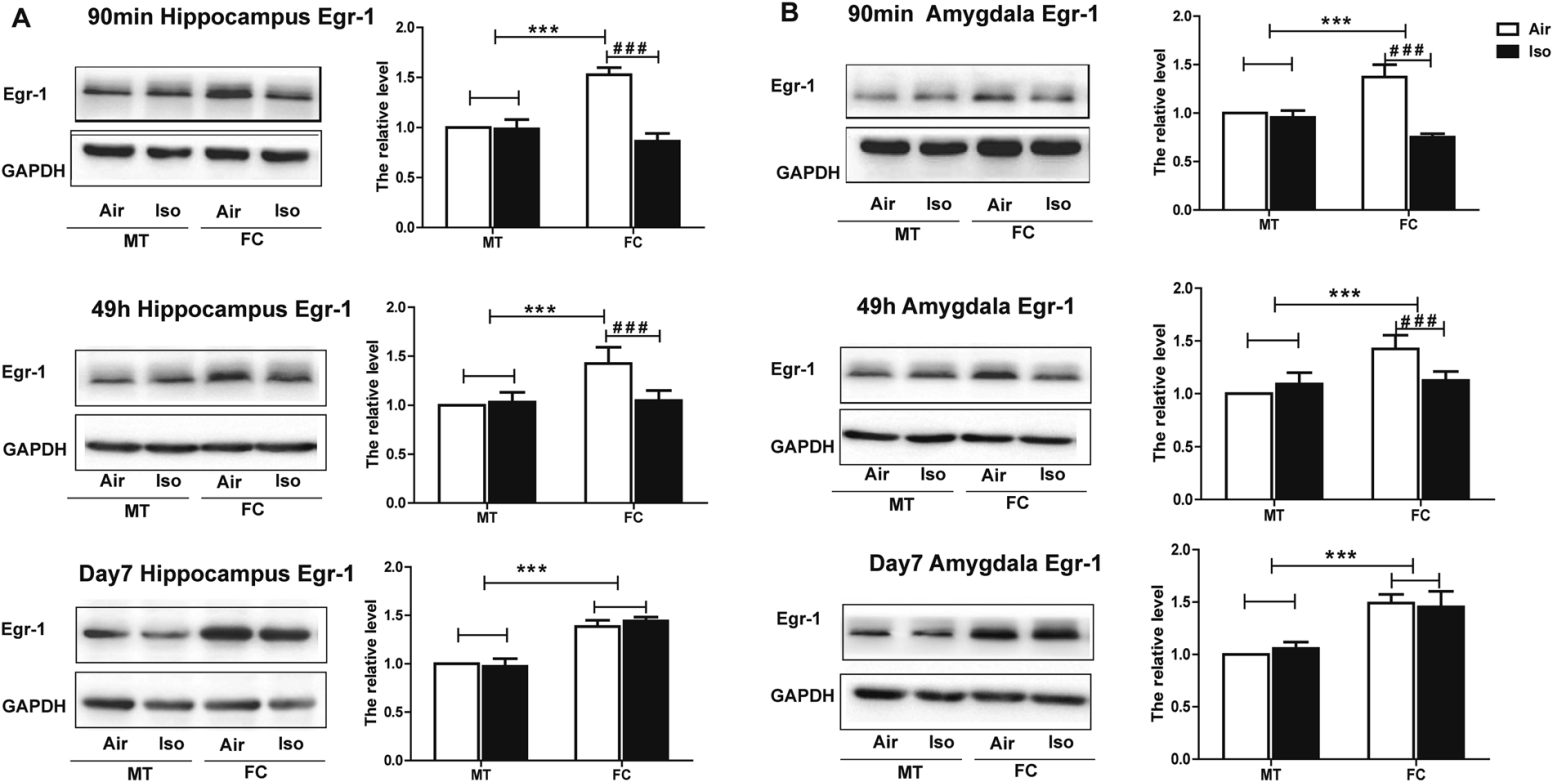
1 h isoflurane exposure decreased the expression of Egr-1 stimulated by fear condition. (**A**) In hippocampus, compared to MT groups, FC groups showed significantly higher level of Egr-1 tested after the 90 min, 49 h and 7 days post conditioning tone fear memory tests, which were impaired by isoflurane. 7 days after FC training, the inhibited Egr-1 expression in the FC group recovered to a similar level as in the Air group. (**B**) In amygdala, similar adverse effect of isoflurane on Egr-1 can be seen. All data are presented as mean ± SEM, n = 6 for each group. ***P < 0.001 compared with MT group; ^###^ *P* < 0.001 compared with Iso group.

Similar change in Egr-1 expression was also observed. As shown in Fig. 4, FC training increased Egr-1 expression level in both hippocampus (Two-way ANOVA, 90 min post condition: F = 49.00, *P* < 0.0001; 49 h post condition: F = 22.79, *P* = 0.0001; post 7-day test: F = 369.2, *P* < 0.0001 Fig. 4A) and amygdala (Two-way ANOVA, 90 min post condition: F = 7.656, *P* = 0.0119; 49 h post condition: F = 35.34, *P* < 0.0001; 7 days post condition: F = 145.4, *P* < 0.0001 Fig. 4B) compared with mock training mice. The increased Egr-1 expression level in FC group was significantly inhibited 90 min and 49 h after FC training in both hippocampus (Two-way ANOVA, 90 min post condition: F = 139.0, *P* < 0.0001; 49 h post condition: F = 14.44, *P* = 0.0012, Fig. 4A) and amygdala (Two-way ANOVA, 90 min post condition: F = 118.2, *P* < 0.0001; 49 h post condition: F = 7.189, *P* = 0.0144, Fig. 4B). 7 days after FC condition, the inhibited Egr-1 expression in FC group recovered to similar level as the Air group (Fig. 4A,B).

### Calcineurin inhibitor rescued the fear memory suppression and calcineurin activity enhancement induced by isoflurane

**Figure 5:**
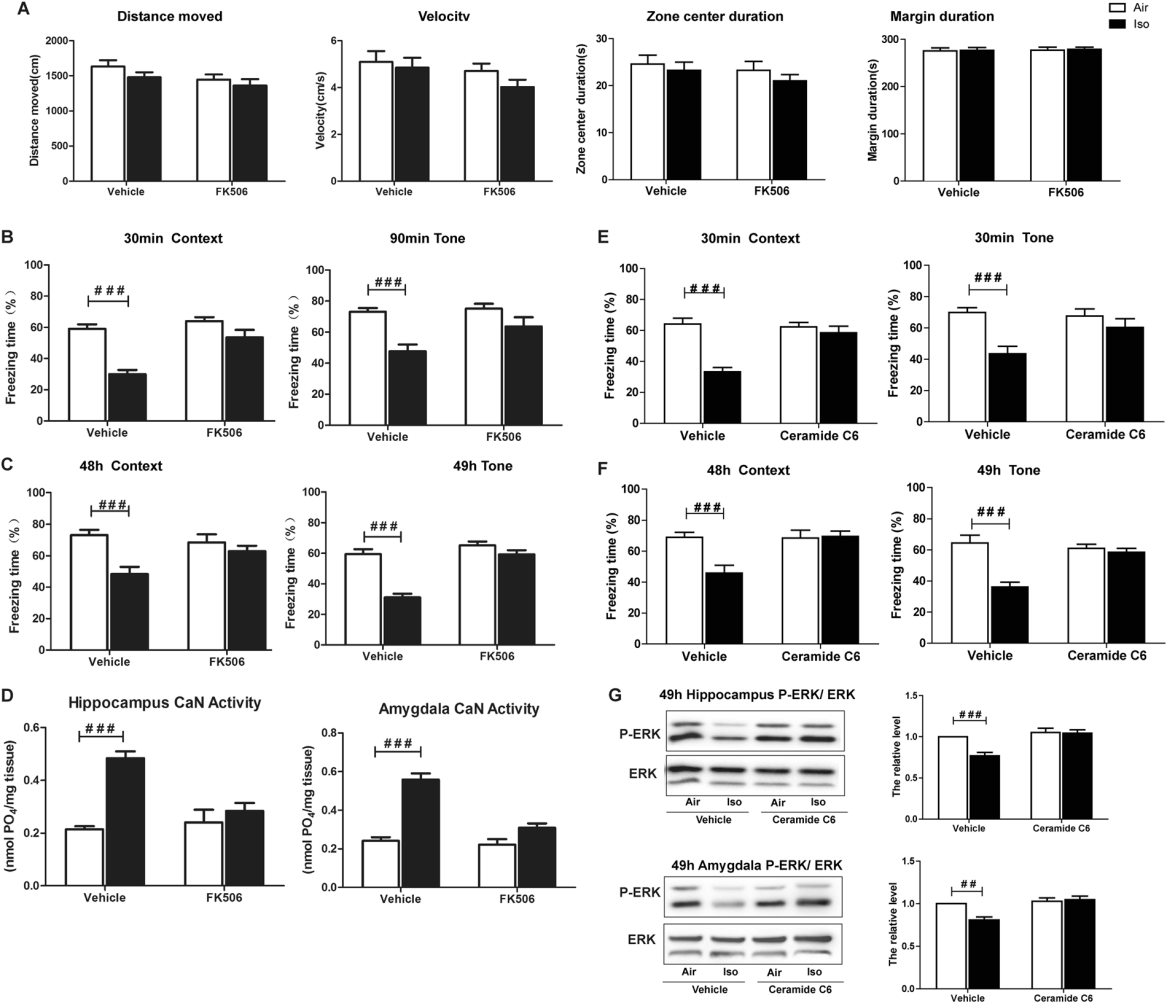
Injection of FK506 and ceramide C6 reversed fear memory impairment and the change of CaN and p-ERK induced by isoflurane. (**A**) Mice from all groups showed similar distance, move velocity, center and margin duration in a 5 min-open field test before fear condition training. 1.3% isoflurane exposure for 1 h significantly decreased the freezing time (%) of mice in both context and tone fear memory test, for both short term (**B**) and long term (**C**), which were rescued by FK506 injection. (**D**) Injection of FK506 attenuated isoflurane anesthesia-induced CaN activity increase. (**E** and **F**) Injection of ceramide C6 rescued isoflurane anesthesia-induced short-term and long-term fear memory impairment. (**G**) Injection of Ceramide C6 before isoflurane exposure attenuated isoflurane anesthesia-induced P-ERK decrease in hippocampus and amygdala. Air + Vehicle *vs*. Iso + Vehicle, ^###^ *P* < 0.001.

To identify the specific role of CaN in the isoflurane-induced fear memory impairment, mice were injected intraperitoneally with CaN antagonist FK506 (5 mg/kg) at the end of 1 h isoflurane exposure according to a previous study^14^. To eliminate the possibility that FK506 modifies behavior of mice, an additional 5 min-open field test was performed before fear condition training. As shown in Fig. 5A, distance and move velocity did not differ among the 1.3%isoflurane groups and air groups with or without FK506 administration. The center and margin duration, which usually reflects the anxiety level of animals, also did not differ among all the groups.

As shown in Fig. 5B to Fig. 5C, isoflurane significantly reduced freezing time in both short-term memory tests (30 min and 90 min post conditioning) (Two-way ANOVA, Context: F = 34.41, *P* < 0.0001; Tone: F = 18.97, *P* < 0.0001) and long-term memory tests (48 h and 49 h post conditioning) (Two-way ANOVA, Context: F = 13.15, *P* = 0.0007; Tone: F = 45.80, *P* < 0.0001). However, FK506 injection after isoflurane exposure could reverse the decreased freezing time caused by isoflurane in both short-term (Two-way ANOVA, Context: F = 17.70, *P* = 0.0001; Tone: F = 4.53, *P* = 0.0389) and long-term memory tests (Two-way ANOVA, Context: F = 5.22, *P* = 0.0273; Tone: F = 25.84, *P* < 0.0001), suggesting CaN inhibition rescued the fear memory suppression induced by isoflurane.

To confirm whether FK506 injection inhibited CaN activity, we tested that at 8 h post isoflurane exposure when CaN activity peaked. As shown in Fig. 5D, there was a significant interaction between the group (Air vs. Iso) and treatment (Vehicle vs. FK506) (Two-way ANOVA, hippocampus: F = 24.33, *P* = 0.0002; amygdala: F = 60.39, *P* < 0.0001) in term of CaN activity and CaN activity returned to normal level when administrating FK506 after isoflurane exposure (Two-way ANOVA, hippocampus: F = 7.452, *P* = 0.0148; amygdala: F = 26.74, *P* < 0.0001).

In order to further clarify the role of P-ERK under specific circumstance, we injected P-ERK activator ceramide C6 i.c.v. bilaterally(50 nM) as previously described^15^ before isoflurane exposure using a separate group of mice, which rescued short-term (Fig. 5E) and long-term fear memory impairment (Fig. 5F), suggesting that P-ERK is involved in the iso-treatment behavioral paradigm. We also assessed the effect of ceramide C6 injection on change of p-ERK at the end of fear memory test. As shown in Fig. 5G, the reduction of ERK phosphorylation in both hippocampus and amygdala induced by isoflurane exposure were reversed by Ceramide C6 injection (Two-way ANOVA, hippocampus: F = 18.01, P = 0.0004; amygdala: F = 14.97, *P* = 0.0009).

### FK506 injection mitigated isoflurane-induced reduction of p-ERK/ERK and Egr-1 expression in the hippocampus and amygdala

**Figure 6:**
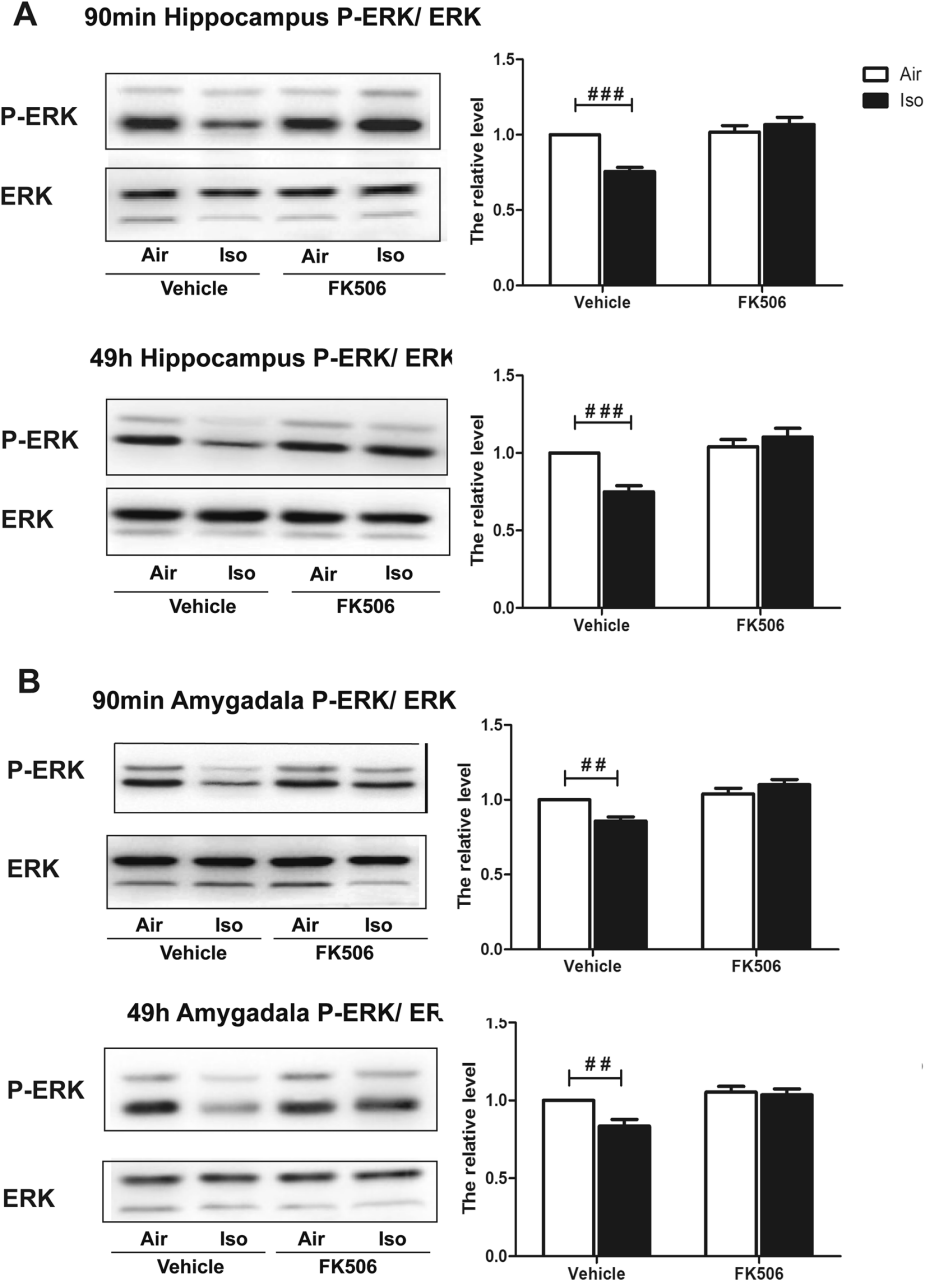
Injection of FK506 after isoflurane exposure attenuated isoflurane anesthesia-induced P-ERK decrease of mice. (**A**) In the hippocampus, FK506 reversed the reduced P-ERK/ERK by isoflurane in the vehicle groups detected after the short-term and long-term fear memory tests; (**B**) In the amygdala, FK506 reversed the reduced p-ERK/ERK by isoflurane in the vehicle groups detected after the short-term and long-term fear memory tests. ^##^ *P* < 0.05, ^###^ *P* < 0.001.

We further assessed effects of FK506 on isoflurane-induced change of p-ERK and Egr-1 expression in the hippocampus and amygdala at the end of short-term and long-term tone fear memory tests, which were performed 90 min and 49 h post FC training, respectively. As shown in Fig. 6A, there was a significant interaction between the group (Air vs. Iso) and treatment (Vehicle vs. FK506) (Two-way ANOVA, 90 min post condition: F = 16.62, *P* = 0.0005; 49 h post condition: F = 13.89, *P* = 0.0012) in term of p-ERK/ERK in the hippocampus and p-ERK/ERK returned to normal levels when administrating FK506 after isoflurane exposure (Two-way ANOVA, 90 min post condition: F = 20.85, *P* = 0.0002; 49 h post condition: F = 21.97, *P* = 0.0001) while there was no significant change of total ERK. In accordance with hippocampus, the reduction of ERK phosphorylation in the amygdala induced by isoflurane exposure was also reversed by FK506 injection (Two-way ANOVA, 90 min post condition: F = 23.73, P < 0.0001; 49 h post condition: F = 12.52, *P* = 0.002 Fig. 6B).

**Figure 7:**
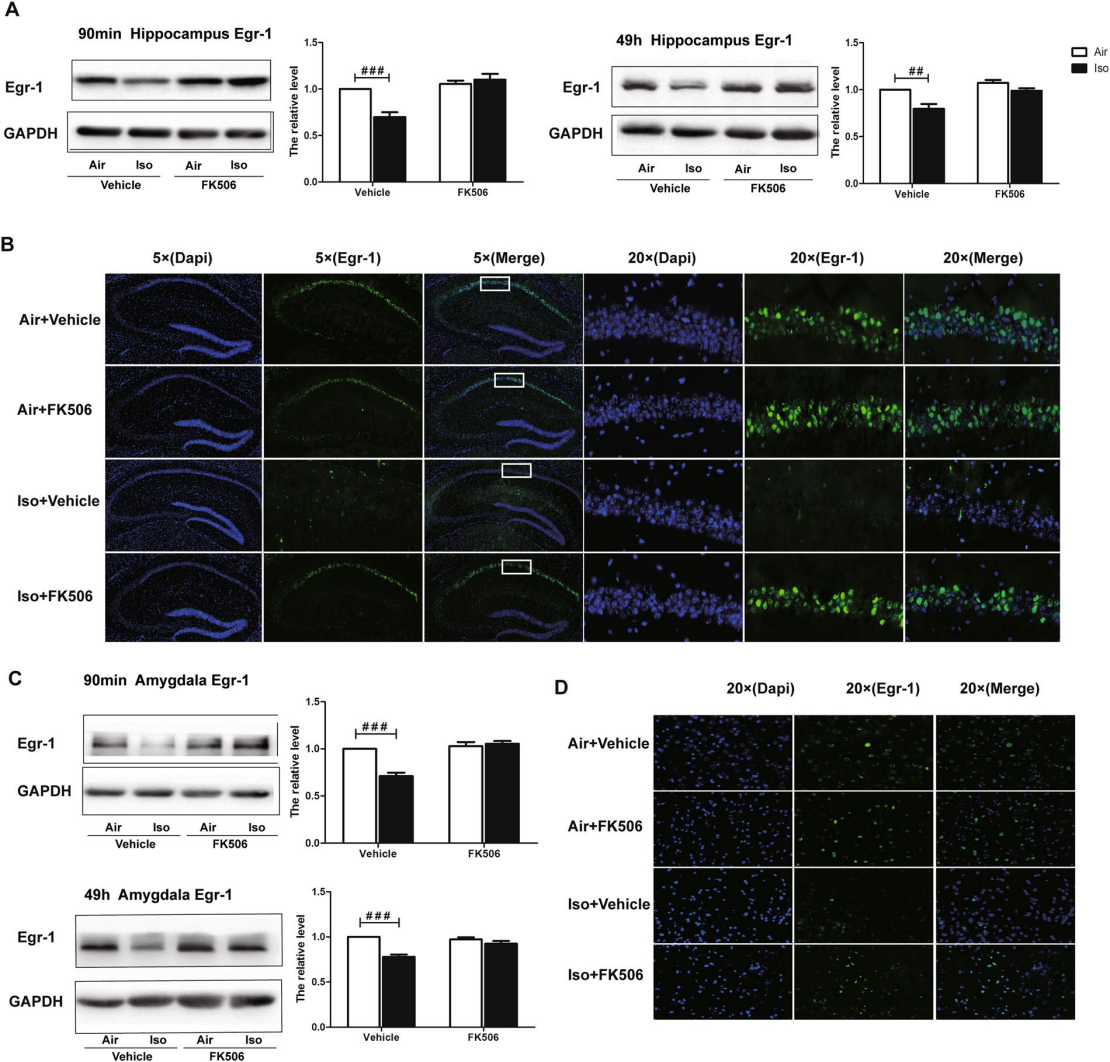
Injection of FK506 after isoflurane exposure attenuated isoflurane anesthesia-induced Egr-1 expression. (**A**) In the hippocampus, FK506 reversed the reduced Egr-1 expression by isoflurane in the vehicle groups detected after the short-term and long-term fear memory tests; (**B**) 5× immunofluorescence pictures show Egr-1 was mainly located in CA1 region of hippocampus, and 20× pictures depict the same alteration of Egr-1 among four groups with western blot; (**C**) In the amygdala, FK506 reversed the reduced Egr-1 expression by isoflurane in the vehicle groups detected after the short-term and long-term fear memory tests; (**D**) 20× immunofluorescence pictures depict the same alteration of Egr-1 in the amygdala among four groups with western blot. ^##^ *P* < 0.05, ^###^ *P* < 0.001.

Meanwhile, similar effects of FK506 on Egr-1 expression in both hippocampus (Two-way ANOVA, 90 min post conditioning: F = 25.75, *P* < 0.0001; 49 h post condition: F = 16.54, *P* = 0.0006 Fig. 7A) and amygdala (Two-way ANOVA, 90 min post condition: F = 31.93, *P* < 0.0001; 49 h post conditioning: F = 6.564, *P* = 0.0182 Fig. 7C) were observed. In addition, immunofluorescence staining further confirmed results from the Western Blot in the hippocampus (Fig. 7B) and the amygdala (Fig. 7D) and showed that the Egr-1 expression change in hippocampus mainly occurred in the CA1 region (Fig. 7B).

## Discussion

Memories can be divided into neutral and emotional memory according to the arousal and valence dimension of events. Compared to neutral memory, emotional memory is stronger and longer-lasting, thus constitutes the core of our personal history^16^, that is why we focused on the influence of isoflurane on fear memory, an important part of the negative emotional memory. Investigating the molecular signal pathway involved in this effect helps to develop potential target to treat POCD. In addition, clarifying how isoflurane works on fear memory may provide novel prevention or treatment clues for emotion associated disorders, like Post Traumatic Stress Disorder (PTSD). We may treat patients experiencing stressful events, like awake craniotomy and other invasive therapies, with isoflurane or similar agents preconditioning to avoid negative emotional memory formation.

Classical FC is a common and typical emotional memory paradigm for rodents, which can distinguish between short- and long-term memory depending on the time point of test. The hippocampus was found to have a selective role in fear to contextual stimuli, while fear memory of auditory tone is dependent on amygdala^13^. In order to elucidate the effect of isoflurane on fear memory at different time and in different brain region, we chose FC as the behavior model in our study.

Isoflurane exposure has been shown to block subsequent formation of fear memory in previous studies^3,4,17^. We have used the same behavior protocol as described by Saab BJ *et al*.^18^ showing that a 1.3% isoflurane exposure for 1 h impaired both short-term and long-term contextual and tone fear memory formation. Considering non-associative fear learning is correlated with the intensity of the electric shock, therefore, nociception was studied in a separate group of mice using the tail flick assay before FC traning^19^ (Supplemental Information Fig. 1). Thus, we could ensure that all groups perceive the same strength of stimulus. Furthermore, studies^20^ have demonstrated that anesthesia-induced hypothermia can directly inhibit ERK activation resulting in decreased expression of ARC, another transcription factor critical for memory consolidation, therefore, the rectal temperature of experimental mice was monitored during isoflurane exposure and at the time of brain tissue harvesting, to get rid of hypothermia. In addition, to ensure that hypoxia was not a contributing factor to memory deficit in the experimental paradigm, arterial blood gases were analyzed in separate groups of mice 5 min and 1 h after isoflurane anesthesia. Hypoxia did not occur and similar values for pH, concentration of bicarbonate, and partial pressure of carbon dioxide and oxygen were obtained (See Supplemental Information Table 1).

In our study, isoflurane failed to change the levels of these molecule and behavior when mice were mock trained in the fear condition chamber. These results suggest that isoflurane repressed contextual and tone fear memory by repressing the activation of CaN/P-ERK/Egr-1 in response to learning but did not affect its baseline levels under mock training conditions. Interestingly, we found that isoflurane did not alter the total CaN protein level in the hippocampus and amygdala. The underlying reason may be that 1 h isoflurane exposure was not sufficient to trigger tremendous variation in CaN expression. In fact, CaN is highly abundant in the hippocampus, striatum, cerebral cortex and cerebellum, constituting 1% of total protein^5,21^. A minor change in CaN expression might not be detected by Western blot analysis. Further advanced method could be used to illustrate this question in future experiment.

To confirm the specificity of FK506 treatment, a separate group of mice were exposed to isoflurane, followed by an i.p. injection with rapamycin (5 mg/kg) in a parallel experimental design. Rapamycin is a macrolide immunosuppressant similar to FK506 in that it inhibits interleukin-2 signaling without the additional inhibition of CaN^22^; thus, any effect elicited by FK506 but not rapamycin is highly likely to be exerted through inhibition of CaN. We found that contextual and tone fear memory in isoflurane-exposed mice which received rapamycin did not differ significantly from the mice which received isoflurane exposure and vehicle (See Supplemental Information Fig. 2). Thus, further confirmed our hypothesis that CaN is involved in isoflurane induced memory impairment.

The notion that p-ERK is a downstream cascade of CaN in the formation of long-term memory has been reported in multiple studies^23,6^. However, CaN is a protein phosphatase with a broad-spectrum of substrates, for example L-type calcium channel, which may also regulate fear memory performance^24^. In the present study, p-ERK activator ceramide C6 injected i.c.v. bilaterally before isoflurane exposure rescued fear memory impairment (Fig. 5E,F) and the reduction of ERK phosphorylation (Fig. 5G), indicating that P-ERK is the actual downstream molecule that are responsible for the behavior impairment rather than a parallel phenomenon stimulated by altered CaN activation.

Our study indicated that isoflurane-induced hippocampal Egr-1 change mainly occurred in the CA1 sub-region, which was consistent with other functional neuronal imaging studies showing that signals in the CA1, instead of other subregions of hippocampus, enhanced in the process of learning and memory^25,26^. In fact, CA1 is the major subregion that has substantial reciprocal connections with the amygdala, which plays a pivotal role in the formation of contextual and tone fear memory^27–29^. Together with the change in the amygdala, Egr-1 expression correlated well with isoflurane-induced contextual and tone fear memory blockade.

FK506 is an immunosuppressant drug used routinely in patients after transplantation. Unlike another CaN inhibitor cyclosporine A, FK506 can easily cross the blood–brain barrier, bind to the ubiquitous intra-cellular protein cyclophilin, and then CaN phosphatase activity is noncompetitively inhibited by the binding of the complex FK-506-cyclophilin^30^. We demonstrated that altered Egr-1 expression was likely mediated by upstream CaN and P-ERK. Enhancement of CaN activities contributed to reduction of ERK phosphoralation and Egr-1 expression, consistent with previous studies^31,32^. Ca^2+^-dependent kinases and phosphatases actively control neuronal processing by forming a tightly regulated balance in which they oppose each other. In this balance, CaN is a critical protein phosphatase whose main function is to negatively modulate learning, memory, and plasticity. It acts by dephosphorylating numerous substrates in different neuronal compartments. CaN is generally activated by Ca^2+^ and calmodulin (CaM) in the brain^33^. Volatile anesthetics including isoflurane can inhibit Ca^2+^ translocation and binding of CaM^34–37^. The Ca^2+^/CaM dependent protein kinase II (CaMKII) and phosphatase CaN are both substrates for Ca^2+^/CaM complex^38^. Perhaps imbalanced activities between CaMKII and CaN led to increased CaN activation after isoflurane exposure. Further study is needed to investigate how isoflurane induces persistent enhancement of CaN activation.

The findings in our study were similar to studies showing that increased CaN activation suppresses memory formation and synaptic plasticity^39–41^. Similarly, inhibition of CaN with FK506 enhances learning and memory in animal models of various neuro-degenerative diseases such as Alzheimer disease, Parkinson disease and multiple sclerosis^42–44^. Together with these findings, our study provides some clue for safer anesthesia care for patients and better postoperative outcomes. Moreover, the results suggested that isoflurane could also be used as a tool to investigate the mechanisms of emotional memory or treat emotion associated disorders.

There are some limitations in our study. Firstly, since there are no specific agonists of CaN for mice by so far, we did not investigate the role of CaN/p-ERK/Erg-1 signaling pathway in the isoflurane-induced blockade of fear memory by enhancing this pathway. Furthermore, we did not use gene knockout mice to further test our hypothesis. This will be carried out in future experiments. In spite of these limitations, we found that exposure of adult mice to 1.3% isoflurane for 1 h negatively regulated short-term and long-term contextual and tone fear memory via increasing the activity of CaN followed by inhibition of ERK phosphorylation and Egr-1expression, in both hippocampus and amygdala.

In conclusion, our study demonstrated that clinical dosage of isoflurane blocked fear memory formation partly through CaN/p-ERK/Erg-1 signaling pathway in both hippocampus and amygdala. These data shed light on molecular mechanisms of amnesia effect of isoflurane and make us understand property of isoflurane better.

## Materials and Methods

### Animals

All the experimental procedures in this study were in compliance with the National Institutes of Health guidelines and were approved by the Animal Ethics Committee in ECNU, China. Adult C57BL6/J mice (aged 8–16 weeks) were obtained from SLAC Laboratory Animal (Shanghai, China). The mice were housed in the animal center of Shanghai Jiao Tong University School of Medicine with free access to food and water. The room temperature was kept in 23 ± 2 °C with a 12-h light-dark cycle (lights on at 7:00 AM; lights off at 7:00 PM).

### Isoflurane Anesthesia and drug administration

Animals in the anesthesia group received 1.3% isoflurane (Baxter Pharmaceutical Products, Inc., Liberty Corner, NJ) balanced with 2 L/min air/nitrogen (30% oxygen and 70% nitrogen) in a tightly sealed plastic chamber for 1 h. This concentration corresponded to 1 minimum alveolar concentration (MAC) in adult mice^42^. Animals in the control group were identically treated except that no isoflurane was flushed to the chamber. To prevent hypothermia during anesthesia, the floor of the chamber was warmed with a heating blanket to keep the rectal temperature in normal physiologic range. The concentration of isoflurane, oxygen and carbon dioxide in the chamber was continuously analyzed with Datex-Ohmeda Ultima Capnomac anesthesia monitor (Helsinki, Finland). During anesthesia induction, the LoRR was determined by manually tilting the chamber to place the mouse on its back, assessing its ability to right itself. If the mouse remained with at least three paws in the air for more than 30 s, its righting reflex was considered to be lost. Time elapsed from start of isoflurane exposure to LoRR was recorded^45,46^ to describe the latency of LoRR. After isoflurane or air treatment, mice were taken from the gas chamber and placed in a second heated clear acrylic chamber for 1 h to recover from anesthesia.

The guide cannula for microinjection i.c.v. of ceramide C6 was implanted as previously described^47^. Briefly, the mouse was anesthetized with ketamine (100 mg/kg, i.p.), and a guide cannula (26 G, Plastics One, Roanoke, VA) was stereotaxically implanted into both sides of lateral ventricle (AP – 0.4 mm; ML – 1.0 mm; V – 2.0 mm).

Mice were allowed for one week recovery before microinjection. Ceramide C6 was injected 30 min before Air/Iso exposure using a minipump (11 Elite Nanomite) and a Hamilton syringe connected to the infusion cannula (33 G, Plastics One) by a polyethylene tubing. 25 nM Ceramide C6 or vehicle at 2 μl volume was administered into both sides at a rate of 0.5 μl/min. After completion of the microinjection, the infusion cannula remained inside the guide cannula for an additional one minute and was then slowly withdrawn to avoid back flow. All injected mice were then randomly assigned to air or isoflurane exposure (n = 14/group).

### Open field test

Before behavioral experiments, all mice were handled daily for 10 min for a minimum of 1 week to reduce acute stress reactions^43^. A 5-min open field test was performed in an open field apparatus (40 cm × 40 cm × 40 cm) with a EthoVision XT 8.5 system (Noldus Information Technology bv, Netherlands) to assess the basal locomotor activity and anxiety level of mice after isoflurane exposure and 1 h recovery time. The parameters of move velocity (cm/s), distance (cm), center zone and margin duration (s) were recorded automatically and analyzed by the EthoVision XT software. Center zone duration was defined as the total time spent not within an 11 cm-beam margin of the arena walls.

### Acoustic Startle Response

At the end of 1 h recovery from Air/Iso exposure, each mouse was placed into sound–attenuating startle chambers to measure startle responses. Following a 5 min habituation period, mice were presented with 25 ms startle stimuli of 7 varying intensities (70–100 dB), with an interstimulus interval of 30 s^48^. Startle stimuli were presented in three blocks each containing 7 stimuli intensities given in a pseudorandom order. The average startle amplitude for each stimuli intensity was calculated from the three blocks.

### Fear conditioning Studies

For fear conditioning experiments, a classical protocol was established as described previously^18^. Briefly, each mouse in either the isoflurane or air group was placed in a conditioning chamber and allowed to explore for 3 min before presentation of a 30 s tone (80 dB, 3,600 Hz). The chamber (50 × 18 × 18 cm) was equipped with a stainless steel grid floor connected to a constant-current shock generator. At the end of the tone, a foot shock (0.7 mA for 0.5 s) was given. The mouse was then allowed to explore the chamber for another 30 s to further associate the context and cue with the shock. Reactivity to the shock during training was estimated by comparing mouse velocity immediately preceding vs. during shock presentation using the following formula: (velocity_shock_ − velocity_pre-shock_)/(velocity_shock_ + velocity_pre-shock_)^49^. To demonstrate that behavior was altered by the training process, two additional Mock Training control groups of mice exposed to isoflurane and air respectively, underwent the same training procedures as above, but received no foot-shock.

30 min after training, STM for context was probed by placing each mouse in the fear-conditioning chamber for 3 min. Fear memory was assessed by measuring the percent of time in freezing behavior for 3 min, defined as “absence of movement except for respiration”. 1 h later, STM for tone was studied by allowing the mice to explore a novel chamber scented with 4% acetic acid. After a 3 min chamber exploration, the same tone (80 dB, 3,600 Hz) was presented for 3 min, during which freezing behavior was measured. 2 days later, LTM for both context and tone was studied in the same way as STM. Seven days later, context and tone fear memory was tested tested in the same way above. The authors were blinded for all behavioral studies.

### Western blotting

CaN protein expression in hippocampus and amygdala were detected at different time points after isoflurane exposure (0 h, 2 h, 4 h, 8 h, 48 h, 7d) and Egr-1, ERK1/2, p-ERK 1/2 in both brain regions were detected at the end of short term and long term fear memory test and 7 days after isoflurane exposure. Mice were decapitated and hippocampus and amygdala were isolated and immediately lysed in ice-cold RIPA buffer supplemented with PMSF, protease and phosphatase inhibitor cocktail solution as described previously by Shen *et al*.^44^. Mice from air exposure control group were treated in the same way. Sample protein concentrations were determined by the BCA assay. Thirty to sixty micrograms of protein from each sample were loaded on a 10% SDS-PAGE gel and electrotransferred onto nitrocellulose blotting membrane (Life Sciences). The membranes were blocked with 5% nonfat milk and incubated with primary antibodies against CaN, Egr-1, ERK1/2, p-ERK 1/2 (Thr202/Tyr204) (antibodies were purchased from Cell Signaling Technology and used at 1:1000 dilution) with gentle agitation overnight at 4 °C. The membranes were incubated with horseradish peroxidase–labeled goat anti-rabbit secondary antibodies for 2 h at room temperature. Immunoreactive protein bands intensity were detected and visualized with a ImageQuant LAS 4000 Mini (GE Healthcare Bio-Sciences, Pittsburgh, PA, USA). Densitometric measurement of band intensity was analyzed using Image J software. To quantify the density of p-ERK and ERK, both of the bands were used to normalize the expression level.

### Immunofluorescence staining

At the end of long term fear memory test, the mice were anesthetized with sodium pentobarbital (50 mg/kg, intraperitoneally) and transcardially perfused with saline, followed by cold 4% paraformaldehyde in phosphate buffered saline^45^. Mice brains were removed, post-fixed, dehydrated and cut into 20 mm thick coronal sections. Sections were simultaneously permeabilized and blocked with 10% donkey serum in phosphate buffered saline supplemented with 0.3% Triton X-100 for 1 h. Sections were incubated with polyclonal rabbit anti-Egr-1, ERK1/2, p-ERK 1/2 (Thr202/Tyr204) (Cell Signaling Technology, 1:100 dilution) overnight at 4 °C, then incubated with Alexa Fluor 488 donkey anti-rabbit for 2 h at room temperature. After immunostaining, the sections were mounted with a drop of anti-fade mounting medium (Beyotime, Shanghai, China) and stored in the dark for observation with confocal microscopy.

### Calcineurin Activity

The hippocampal and amygdala tissues were obtained at different time points (0 h, 2 h, 4 h, 8 h, 48 h, 7d) after isoflurane exposure and processed into protein extracts. The CaN (PP-2B) activity of protein extracts were then assayed with a colorimetric kit (EMD Biosciences, San Diego, CA) employing RII phosphopeptide as substrate according to the manufacturer’s instructions, which is the most efficient and selective substrate known for CaN. The free phosphate released is detected using malachite green according to the manufacturer’s instructions.

### Statistical analysis

The sample size selected for fear-associated memory studies was determined by a pilot study of 14 mice (7 male, 7 female) that demonstrated a deficit in short-term contextual fear memory after exposure to isoflurane for 1 h compared with air-treated controls. In control animals, the mean freezing score was μ_0_ = 69.7% and the SD was δ_0_ = 12.5%. In mice treated with isoflurane, the freezing score was μ_1_ = 55.2% and SD was δ_1_ = 16.2%. The sample size was calculated using a formula where n = (z_1−β_ + z_1−α_)^2^(δ_0_^2^ + δ_1_^2^)/(μ_0_ − μ_1_)^2^ based on a one-tailed test with α value equal to 0.05 and 1-β value equal to 80%. Data are reported as mean ± S.E.M. The protein expression of isoflurane groups was presented as a percentage of those of Air group. An unpaired two-tailed Student’s t test was used to calculate differences between two groups. Differences among multiple groups were analyzed by two-way analysis of variance (ANOVA) with non-repeated measures. The two factors were Air/Iso and MT/FC or Vehicle/FK506. P < 0.05 was considered to be statistically significant. All graphs and statistical analyses were performed using the GraphPad Prism 5 software (GraphPad, La Jolla, CA, USA).

## Electronic supplementary material

Supplementary Material
