# Supplementary material for: Calcineurin/P-ERK/Egr-1 Pathway is Involved in Fear Memory Impairment after Isoflurane Exposure in Mice
Source: Sci Rep. 2017 Oct 24;7:13947. doi: 10.1038/s41598-017-13975-z (PMC5654981; doi:10.1038/s41598-017-13975-z)
Supplement: Supplementary file 1 — Supplementary Material [file 41598_2017_13975_MOESM1_ESM.pdf]

Supplemental Information.

Calcineurin/ P-ERK/ Egr-1 Pathway is Involved in Fear Memory Impairment after Isoflurane Exposure in Mice

Xiaoxuan Yang, Guohui Li, Qingsheng Xue, Yan Luo, Sensen Wang, Yimeng Xia, Lei Zhuang and Buwei Yu

Supplemental information Methods:

Acoustic Startle Response:

The acoustic startle response was measured with a modified device described previously[1]. Startle testing was conducted in a MED-ASR-PRO1 startle testing system (MedAssociates, VT, USA). Mice were placed in a plexiglas cylinder for testing. Acoustic startle stimuli were delivered via a high-frequency speaker, placed at a distance of 15 cm from the testing cylinder. Background noise levels were maintained at 65 dB throughout the experiments. The testing cylinder was mounted on a sensor platform. A piezoelectric accelerometer, attached to the base of the sensor platform, detected and transduced all cage movements, and these were recorded by a computer. The startle amplitude was taken to be the maximal response occurring within 100ms of the presentation of the startle stimulus. Startle responses are in manufacturer's arbitrary units. The speakers, testing cylinder and sensor platform were housed within a sound-attenuated chamber. Mice were initially habituated to acclimate them to the testing environment.

Supplemental information Figure 1.

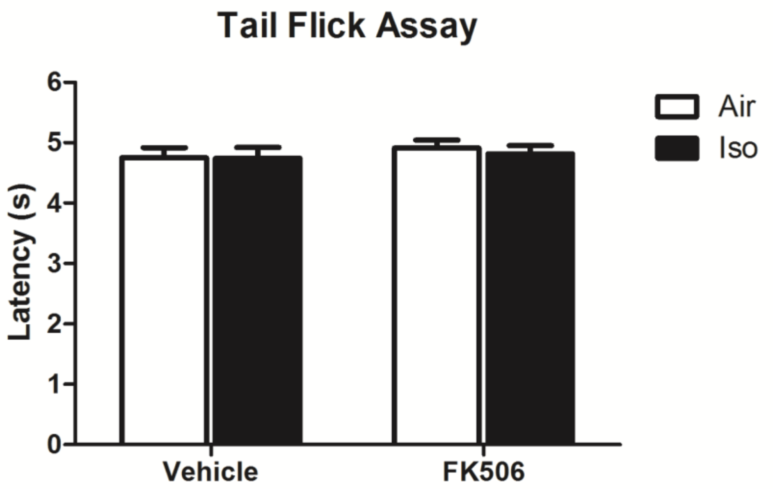

Supplemental information Figure 1. Latency to flick tail away from a hot water bath. Latency to tail flick was not influenced by isoflurane exposure or FK506 injection (n=12 per group).

Supplemental information Figure 2.

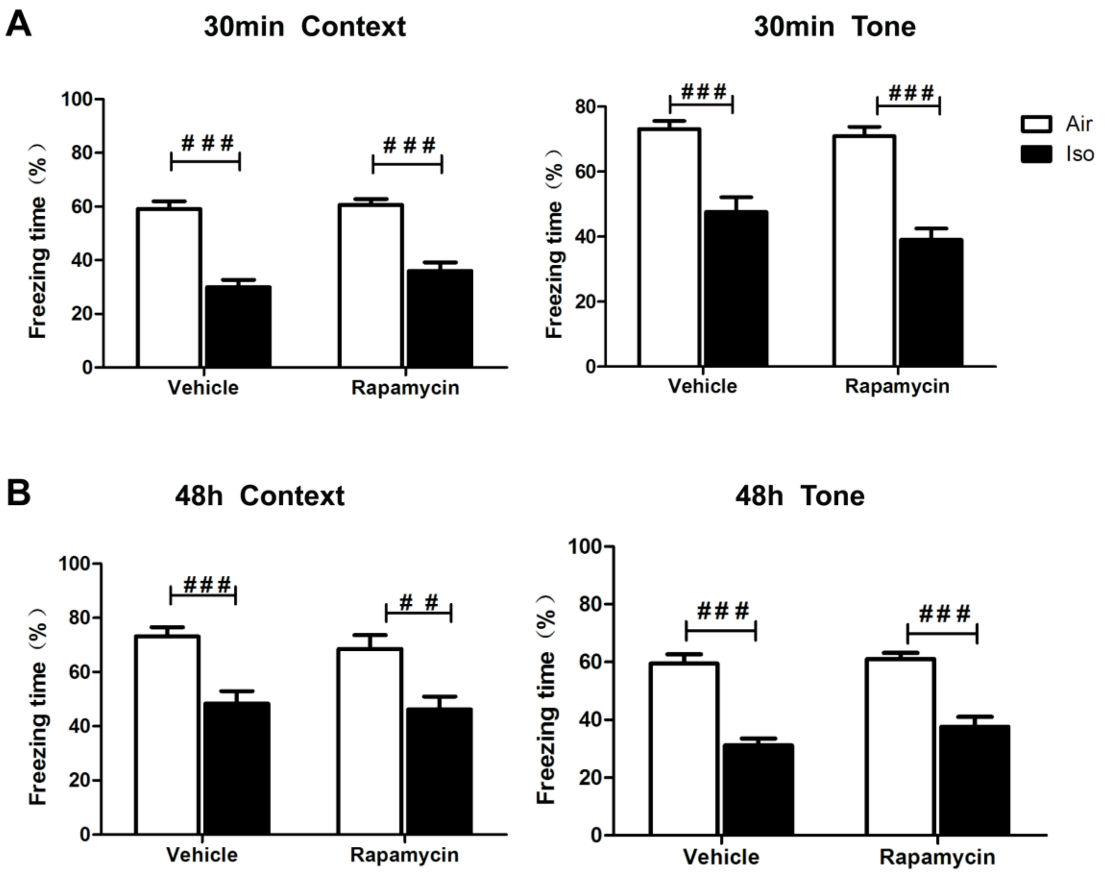

Supplemental information Figure 2. Rapamycin injection can not rescue the context and tone fear memory impairment induced by isoflurane exposure. A. 30min after fear condition, mice exposed to isoflurane exhibited a memory deficit to context and tone, evidenced by lower freezing score compared to air-treated controls

in both vehicle group and rapamycin group. **B.** 48h after fear condition, decreased freezing score in isoflurane exposure group in both context and tone fear memory tests could not be rescued by rapamycin injection, either. (n=12 per group)

Supplemental information Table 1.

Measure	5 min (n=6)	60 min (n=6)	*P* Value
pH	7.308 ± 0.01086	7.331 ± 0.026	0.409
pCO_2 (mmHg)	45.73 ± 3.649	50.78 ± 3.152	0.3196
pO_2 (mmHg)	155.8 ± 13.31	207.5 ± 18.96	0.0498
HCO_3 (mM)	20.63 ± 0.7671	21.62 ± 1.367	0.5444
Rectal Temperature (℃)	36.93 ± 0.1476	36.87 ± 0.1563	0.7629

Supplemental information Table 1. Blood gas analysis and rectal temperature of mice anesthetized with isoflurane for 5min *versus* 60min. Arterial blood gas analysis showed hypoxia did not occur. In addition, similar values for pH, concentration of bicarbonate, and partial pressure of carbon dioxide and oxygen were obtained.

Reference:

1. Sakaguchi, M. *et al.* Impaired spatial and contextual memory formation in galectin-1 deficient mice. *Mol. Brain* **4,** 33 (2011).
